# Military Personals

**Published:** 1918-03

**Authors:** 


### MILITARY PERSONALS

Dr. Herman Johnson of Gowanda has returned from France on account of ill health and is at present stationed at Newport News.

To Cleveland, O., Capt. Wm. B. Reid, Rome.

To Fort Oglethorpe, Capt. Frederick Terwilliger, Spencer., Lieuts. Patrick H. Buckley, Buffalo, Lee A. Hadley, Syracuse.

To Fort Sam Houston, Texas, Lieut. Ralph Robinson, Belmont.

To Hoboken, N. J., Lieut. Eugene Boudreau, Auburn.

To Philadelphia, Pa., Lieut. John H. Watson, Buffalo.

To Rockefeller Institute, Lieut. Edwin Foster, Penn Yan.

To Camp Kearny, Linda Vista, Calif., Lieut. Anthony J. Grace.

To Camp Lee, Petersburg, Va., Lieut. Peter J. Barone, Buffalo.

To Camp MacArthur, Waco, Texas, Capt. Robert R. Mccully, Auburn, Lieut. Hall G. Van Vlack, Forestville.

To Fort Morgan, Ala., Lieut. Maxwell K. Willoughby, Auburn.

To Fort Oglethorpe, Capt. John R. Bradley, Rochester, Lieuts. Chas. J. Brone, Julius Y. Cohen, Frank G. Walz, Buffalo, Wm. W. Carleton, Waterloo, Henry M. Spefford, Batavia, Floyd P. Breese, Elmira, Harry Wrenker, Rochester, A. C. Smith, Elmira.

Honorably discharged on account of being physically disqualified for active service, Capt. Malcolm E. House, Cuba, Lieut. George R. Stevenson, Penn Yan.

To Camp Joseph E. Johnston, Jacksonville, Fla., Lieut. George S. Skiff, Gainesville.

To Fort Redman, Lieut. Adelbert C. Abbott, Syracuse.

To Morrison, Va., Lieut. Albert A. Getman, Syracuse.

To Rockefeller Institute, Lieut. Arthur W. Hubbard, Wyoming.

To Army Medical School, Washington, D. C., Lieuts. Harry G. Johnson, Earl H. Lermer, Frank H. Long, Harold J. McDonald, Frederick E. Sperry, Buffalo, Frank H. Snyder, Geneva, John P. Sharp, Niagara Falls, Paul S. Persons, Ripley, Benjamin S. Slacer, Rochester.

To Camp Dodge, Des Moines, Iowa, Lieut. Henry W. Duryea, Buffalo.

To Camp Sheridan, Montgomery, Ala., Lieut. Edmund B. Spaeth, Elmira.

To Fort Riley, Lieut. Carl G. Frost, Buffalo.

To Fort Sill, Okla., Capt. Edwin S. Ingersoll, Rochester.

To Hoboken, N. J., Lieuts. Clark V. Fairbanks, Dansville, Milton J. Johnson, Jamestown.

To New York City, Eugene H. Beudreau, Auburn.

To Takoma Park, D. C., Capt. Edward Dowdle, Oswego.

To Williamsbridge, N. Y., Capt. James C. Davis, Rochester.

Lt. Col. E. B. Hardy of the Army Medical Corps, addressed the last meeting of the Medical Union, at the Iroquois, Buffalo.

Drs. Salvator Chas. Lopacone, Samuel Grienstein and Hiram Samuel Yellen of Buffalo have been commissioned lieutenants in the M. R. C. Dr. Louis C. Howes of Buffalo, has been commissioned lieutenant in the Dental Reserve Corps.

Dr. Harry Lee Brown, Surgeon, U. S. N., returned to this country late in Jan. and visited in Buffalo.

Major Edwin L. Beebe of Buffalo, has been honorably discharged from service, for physical reasons held to disqualify him for active service in France. He was commissioned captain in 1904, major in 1914, was transferred from the old 74th to the 12th in 1916 at the time of the call to Texan border service and was with the latter regiment throughout its stay at the border. He was again transferred to the 74th and went with it to Camp Wadsworth, last fall. We regret that Major Beebe has been disappointed in regard to foreign service but congratulate him on his honorable record and trust that he will be able to do valuable work at home, both in private life and in the home guard.

Lt. Wilfred H. Baines of Buffalo is in service in France.

Lt. Frank M. Ende of Buffalo is serving in a Red Cross Hospital in Glasgow.

Our Associate Editor for Canada, D. J. G. Adami of Montreal, is Medical Historical Recorder of the Canadian Expeditionary Forces, with headquarters in London.

